# Outpatient Parenteral Antimicrobial Therapy in Asia: Evolution, Models, and Challenges over the Past Decade—A Narrative Review

**DOI:** 10.3390/antibiotics15070689

**Published:** 2026-07-15

**Authors:** Hsien-Po Huang, Po-Hsiu Huang, Wei-Hsuan Huang, Chia-Wei Liu, Chien-Hao Tseng, Hsiu-Wen Wang, Chia-Hsin Cheng, Chun-Mei Ho, Po-Yu Liu, Ting-Kuang Yeh

**Affiliations:** 1 Division of Infectious Diseases, Department of Internal Medicine, Taichung Veterans General Hospital, Taichung 407, Taiwan; 2 School of Medicine, College of Medicine, National Yang Ming Chiao Tung University, Taipei 112, Taiwan; 3 Graduate Institute of Biomedical Engineering, National Chung Hsing University, Taichung 402, Taiwan; 4 Institute of Molecular Biology, National Chung Hsing University, Taichung 402, Taiwan; 5 Infection Control Center, Taichung Veterans General Hospital, Taichung 407, Taiwan; 6 Department of Post-Baccalaureate Medicine, College of Medicine, National Chung Hsing University, Taichung 402, Taiwan; 7 Doctoral Program in Translational Medicine, Rong Hsing Translational Medicine Research Center, National Chung Hsing University, Taichung 402, Taiwan

**Keywords:** outpatient parenteral antimicrobial therapy, Asia, hospital-at-home

## Abstract

**Background**: Outpatient Parenteral Antimicrobial Therapy (OPAT) has long been established as a safe and cost-effective alternative to inpatient care in Western healthcare systems. In contrast, its implementation across Asia has historically been fragmented and unstructured. A landmark multinational survey in 2017 identified OPAT in Asia as a “missed opportunity,” citing the lack of systematic oversight, standardized protocols, and outcome monitoring systems. **Methods**: This narrative review examines the evolution of OPAT in Asia over the past decade, synthesizing evidence from regional studies, national policies, and implementation models. **Results**: The findings demonstrate a clear shift from ad hoc infusion practices toward structured, multidisciplinary OPAT programs in several healthcare systems. These systems increasingly integrate antimicrobial stewardship principles, dedicated care teams, and reimbursement mechanisms. In parallel, innovative models, including hospital-at-home services and elastomeric pump–based continuous infusion, have expanded treatment feasibility while enabling the use of narrower-spectrum antibiotics. Despite these advances, substantial heterogeneity persists. Key barriers include the absence of unified national guidelines, limited monitoring infrastructure, workforce constraints, and regulatory restrictions in certain settings. Resource-limited regions continue to face challenges related to financing, laboratory capacity, and digital health integration. **Conclusions**: Overall, OPAT in Asia is transitioning from an underrecognized and inconsistently delivered service to an increasingly structured component of infectious disease care. Future priorities include standardized governance, integration with antimicrobial stewardship programs, sustainable reimbursement, digital outcome monitoring, and region-specific evidence on antimicrobial stability and safety.

## 1. Introduction

Outpatient Parenteral Antimicrobial Therapy (OPAT) is characterized as the administration of intravenous anti-infective agents within an ambulatory framework, serving as either a strategic transition from acute inpatient care or a primary outpatient intervention based on clinical necessity [1]. Historically, the conceptualization of OPAT gained traction in the United States during the 1970s as a pragmatic solution for medically stable patients who required parenteral therapy but lacked sufficient insurance coverage [2]. Healthcare administrators rapidly identified that this modality offered significant cost-containment advantages for patients capable of receiving treatment outside the hospital. OPAT offers multiple clinical, economic, and systemic advantages compared with inpatient treatment. Economically, OPAT is associated with substantial cost savings, primarily driven by reductions in inpatient bed utilization and hospitalization costs. Although outpatient expenses such as co-payments, transportation, and home care services may partially offset these savings in some healthcare systems, the overall cost–benefit remains favorable, particularly when indirect benefits, including improved productivity and reduced caregiver burden, are considered [3]. From a healthcare system perspective, OPAT improves patient flow, increases hospital bed capacity, and enables more efficient allocation of limited resources, allowing hospitals to focus on acute and critically ill patients. Clinically, OPAT has been shown to be safe and effective, including for infections caused by antimicrobial-resistant organisms, while reducing the risk of hospital-acquired infections and their associated costs [3]. Beyond financial and system-level benefits, OPAT enhances patient quality of life by allowing treatment in a home-based or outpatient setting, minimizing stress and disruption to daily routines, promoting emotional well-being, and supporting a more flexible and patient-centered care experience [4].

Despite the compelling evidence supporting its efficacy, the global implementation of OPAT remains heterogeneous. Since its initial description in 1974, OPAT has become well established in Europe, North America, and Australia, whereas published data from South and Central America, Africa, Oceania, and parts of Asia remain comparatively limited, suggesting that implementation in these regions may be less extensive or less well characterized than in early-adopter settings [5]. In Australia, the “Hospital-in-the-Home” (HITH) initiative has successfully operationalized OPAT for over two decades [6]. Similarly, Singapore integrated OPAT into its public healthcare infrastructure in 2002, utilizing specialized multidisciplinary teams to manage patients in dedicated outpatient centers [7]. While many patients in these regions leverage 24 h infusion technology for self-administration or community-based nursing support, significant challenges persist in models that lack dedicated staff continuity or robust outcome registries. Despite the clinical benefits of OPAT, its implementation remains inconsistent globally, particularly across Asia. A landmark study in 2017 by Fisher et al. involving 171 healthcare facilities across 17 Asian countries highlighted a stark disparity in service provision [8]. The study revealed that while 57% of facilities administered outpatient parenteral antibiotics, only 3% (excluding Singapore) operated comprehensive services with specialist oversight. Singapore stands as a notable exception, having successfully implemented comprehensive OPAT services on a national scale. In contrast, most other regions rely on an “ad hoc” approach, where treatments are administered through individual private clinics or emergency department infusion services without centralized oversight. Furthermore, the survey identified a critical deficit in data management: only eight services maintained a database for tracking outcomes, and only three of those utilized electronic systems [8]. This lack of structured oversight represents a “missing opportunity” to optimize patient outcomes and reduce the burden on acute care hospital beds. A decade has elapsed since the initial assessment by Fisher et al. The present study, therefore, aims to re-evaluate the current landscape of outpatient parenteral antimicrobial delivery in Asia, focusing specifically on Asian healthcare systems with published OPAT literature. By identifying evolving trends and persistent gaps, this research seeks to outline strategic avenues for the international expansion and systematic refinement of these delivery models.

## 2. Methods

We conducted a targeted narrative review of OPAT in Asian healthcare systems. We searched PubMed, together with the official guideline and policy repositories of the Centers for Disease Control and Prevention (CDC) and World Health Organization (WHO), from database inception to 31 May 2026. The search combined the following terms using Boolean operators: (“outpatient parenteral antimicrobial therapy” OR “OPAT” OR “outpatient parenteral antibiotic therapy”) AND (“Asia” OR the name of each individual Asian country/region). Reference lists of key articles, guidelines, and reviews were manually screened to identify additional sources. Grey literature, including national health-authority policy documents, reimbursement announcements, and clinical practice guidelines (e.g., the Singapore Ministry of Health elastomeric pump guidance and the Hong Kong IMPACT guideline), was eligible given its direct relevance to OPAT policy and implementation.

Eligibility criteria: We included peer-reviewed studies (systematic reviews, randomized or cluster-randomized trials, observational and epidemiological studies, implementation and feasibility studies, and economic evaluations), conference abstracts and authoritative guideline or policy documents reporting on OPAT delivered in one or more Asian healthcare systems. We excluded studies that did not address OPAT, those reporting exclusively on non-Asian populations without extractable Asian data, and non-English publications without an English-language full text or abstract. Two infectious diseases physicians independently screened titles, abstracts, and full texts, with disagreements resolved by discussion and consensus.

**Figure 1:**
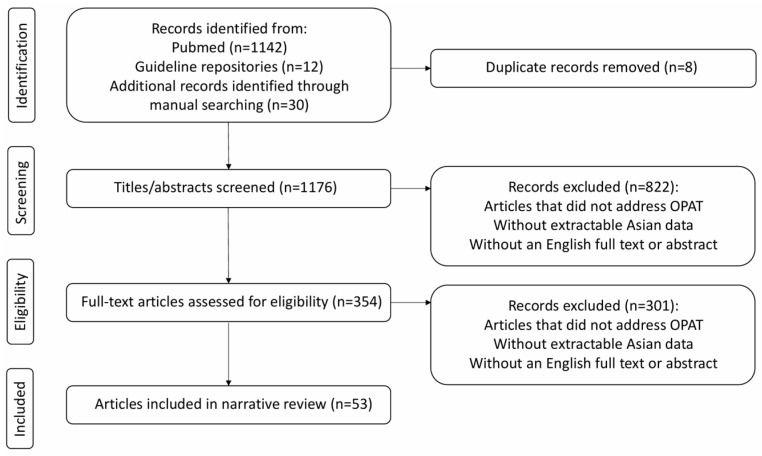
Study identification and selection flow diagram for the targeted narrative review.

A total of 1184 records were identified, of which 1176 were screened after de-duplication, 354 full texts were assessed for eligibility, and 53 sources were included (Figure 1). Healthcare system-specific evidence was synthesized thematically according to service model, governance structure, reimbursement mechanism, antimicrobial stewardship integration, and reported clinical or economic outcomes. Characteristics of the included studies are summarized in Supplementary Table S1.

## 3. Results

**Figure 2:**
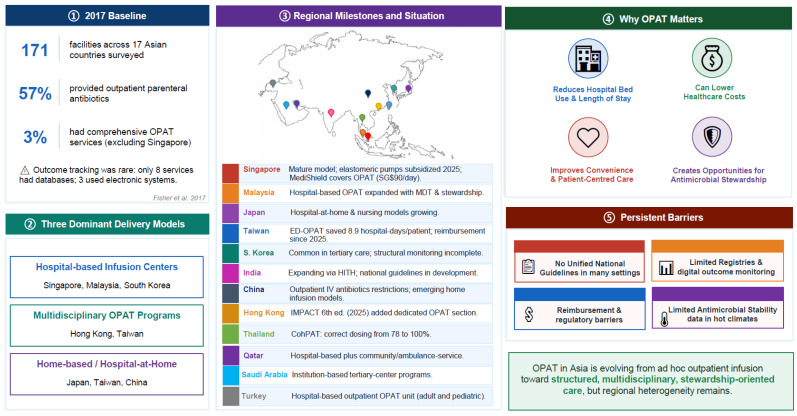
Overview of the OPAT delivery models in twelve Asian healthcare systems. Different-colored labels represent different healthcare systems [8].

A key observation from this review is the emergence of three dominant OPAT delivery models in twelve Asian healthcare systems: (1) hospital-based infusion center models (e.g., Singapore, Malaysia, South Korea), (2) integrated multidisciplinary OPAT programs with centralized coordination (e.g., Hong Kong, Taiwan), and (3) home-based or hospital-at-home (HaH) models supported by visiting nursing services (e.g., Japan, Taiwan, China) (Figure 2 and Table 1). These models reflect adaptations to local healthcare infrastructure, reimbursement mechanisms, and demographic pressures, particularly aging populations and increasing demand for efficient healthcare resource utilization. While no single model is universally applicable, successful programs share core components, including multidisciplinary teams, standardized patient selection criteria, and robust monitoring systems [7].

### 3.1. Singapore

As a pioneer in Asia, the evolution of OPAT in Singapore began in 2002, leading to the decentralized adoption of such programs across most public hospitals. While the primary clinical model involves patients visiting outpatient centers, there is established provision for home-based care specifically tailored for those with mobility constraints [38]. A key strength of the Singaporean model is the collaborative data sharing among academic hospitals, which facilitates a comprehensive national overview of service utilization. By maintaining a combined outcomes registry, the system enables a holistic evaluation across various provider sites. Singapore’s national program has matured to align with all components of the recognized OPAT bundle [4,7]. Orthopedic conditions accounted for 40% of all patients, while vancomycin was the most commonly prescribed antibiotic, comprising 34% of usage. The readmission rate in Singapore was 8.9%, and OPAT-related complications were infrequently identified as contributing factors. OPAT resulted in an estimated cost savings of Singapore dollar (SGD) 207,200 across 51 patients in 2005 [7]. A pivotal shift in the Singaporean landscape occurred in April 2025, when the Ministry of Health officially recommended subsidies for elastomeric infusion pumps. This policy enables patients to receive continuous antimicrobial infusions in non-inpatient settings, including both OPAT clinics and at home [9]. Furthermore, Singapore has expanded its national MediShield Life insurance to include coverage for OPAT services, with a claim limit of 90 Singapore dollars per day [10].

### 3.2. Malaysia

In Malaysia, OPAT has gradually evolved from a specialized hospital-based service toward broader implementation in public hospitals, with increasing recognition of its role in antimicrobial stewardship over the past decade. Formally established in 2016 at Hospital Sungai Buloh, the service initially served as a pilot for public infectious disease management, proving the model’s safety and feasibility in the local context [11]. Clinical data indicates that the most frequent indications are bacteremia (53.5%) and intra-abdominal abscesses (30.2%), with Ceftriaxone (34.9%) and Ceftazidime (25.6%) being the primary therapeutic agents. While the clinical success rates remain high with a manageable readmission rate of approximately 9.3% among 43 patients between 2016 and 2017, challenges persist regarding the standardization of protocols and the accessibility of national guidelines across rural states [11]. Historically, Hospital Sungai Buloh served as the sole provider of OPAT services in Malaysia. Expansion of the program to several major hospitals occurred only after the COVID-19 pandemic. The Malaysian OPAT system largely follows a hospital-based model, whereby patients attend the hospital daily to receive outpatient intravenous antimicrobial therapy. Administration is generally performed either via peripheral venous cannulation or through continuous infusion using an elastomeric pump connected to a peripherally inserted central catheter [12].

In Malaysia, the Ministry of Health has implemented OPAT across various public hospitals, primarily utilizing an infusion center model. Under this framework, patients visit the facility at designated times to receive their intravenous treatments rather than staying as inpatients. The delivery of this service relies on a multidisciplinary team, which typically includes infectious disease physicians, clinical pharmacists, nurses, and assistant medical officers. This team is responsible for managing the entire scope of care, from defining patient selection criteria and clinical protocols to overseeing treatment courses and administrative processes. To ensure a consistent standard of care nationwide, healthcare providers involved in the program participate in regular training sessions and workshops [12].

### 3.3. Japan

The evolution of OPAT in Japan reflects a unique intersection of demographic necessity and antimicrobial stewardship. However, the field still lacks a unified national protocol. Currently, there is a growing shift from clinic-based OPAT models toward homecare supported by visiting nursing services, a trend driven by Japan’s super-aged society and the COVID-19 pandemic [13,14]. These “hospital-at-home” services, provided through regular visits and emergency house calls, encompass medication prescription and the administration of intravenous or subcutaneous antibiotics [13]. Unlike many regions that rely on long-acting, broad-spectrum agents (such as ceftriaxone), the Japanese model is characterized by the common use of elastomeric infusion pumps for continuous administration. This technology facilitates the outpatient use of narrow-spectrum antibiotics, such as cefazolin and penicillin G, which would otherwise require frequent daily dosing [13]. This approach significantly enhances antimicrobial stewardship by preventing the overuse of broad-spectrum alternatives and reducing the selection pressure for resistant bacteria. The estimated reduction in medical expenses was about 87,000 US dollars (USD) over 5.5 years (from July 2012 to December 2017) for 66 patients [13].

### 3.4. Taiwan

Historically, OPAT in Taiwan was managed through the Emergency Department (ED) as a strategy to alleviate boarding and reduce avoidable admissions. ED-initiated OPAT effectively saved an average of 8.9 hospital days and reduced costs by NT$34,367 per patient, without increasing severe adverse events or 14-day readmission rates, demonstrating its potential as a cost-saving alternative to traditional hospitalization [15]. A significant milestone occurred in August 2025, when the National Health Insurance Administration officially launched the national OPAT reimbursement program, which covers patients referred from the emergency department, recently discharged patients, and outpatients [16]. Eligible indications include urinary tract infection, pneumonia, soft tissue infection, osteomyelitis, spinal infection, endocarditis, septic arthritis, postoperative infection, and device-related infection. In addition, to provide physicians with more antimicrobial selection options, portable infusion devices have been included since November, 2025. Unlike the decentralized approach in previous years, the current Taiwanese model emphasizes a multidisciplinary team approach, involving infectious disease specialists, dedicated case managers, and pharmacists to ensure antimicrobial stewardship and patient safety. Furthermore, Taiwan is increasingly integrating “Hospital-at-Home” (HaH) models since 2024, which combine OPAT with visiting nursing services, a shift that has shown good patient satisfaction and significantly lower medical expenditures compared to inpatient care. Moreover, the National Health Insurance Administration (NHIA) introduced an “Early Discharge Model” under the Acute Hospital Care at Home (ACAH) Program in 2026. Inpatients with infectious diseases who have mobility limitations or difficulty accessing medical care may be discharged early and continue therapy at home or in care facilities [16,17,18].

### 3.5. South Korea

In South Korea, OPAT is increasingly used in tertiary care settings, mainly to reduce hospitalization and facilitate outpatient antimicrobial administration. However, current evidence remains largely institution-based, and standardized national OPAT protocols and monitoring systems are still limited. Commonly treated conditions include urinary tract infections (27.3%), respiratory infections (20.8%), and intra-abdominal infections (15.9%). The most frequently prescribed agents are Ertapenem (26.0%) and Ceftriaxone (12.8%), reflecting a reliance on long-acting, broad-spectrum antibiotics to simplify dosing schedules [19]. Recent data indicates that the referral model (where patients are referred to smaller clinics for injections) and the outpatient model (where patients return to the hospital clinic) are the two dominant delivery methods [19]. Despite the high volume of prescriptions, South Korea faces structural challenges. A major concern is the lack of systematic monitoring protocols; studies show that post-prescription follow-up visits are missed in nearly 25% of cases, and laboratory monitoring, which is critical for safety, is inadequate in more than half of the cases [19]. To address these gaps, South Korean scholars have advocated for the rapid integration of OPAT into Antimicrobial Stewardship Programs (ASP) [20].

### 3.6. India

In India, while informal OPAT is practiced in many small clinics, the implementation of structured, evidence-based OPAT remains relatively rare and faces significant cultural and logistical barriers [21]. Recent pilot studies have demonstrated that structured OPAT is not only feasible but can reduce the duration of hospitalization. Urinary tract infections (30%) and gastrointestinal infections (20%) were the most common indications for OPAT. Its implementation shortened hospital stays by an average of two weeks [22]. The pharmacological profile of Indian OPAT is heavily influenced by the high prevalence of Extended-Spectrum Beta-Lactamase (ESBL) producing organisms. Consequently, Ertapenem is frequently the preferred choice for OPAT, especially for treating complicated urinary tract infections, due to its once-daily dosing and efficacy against multidrug-resistant bacteria [23]. Despite these benefits, the widespread adoption of OPAT in India is hindered by the lack of a national standardized protocol and limited infrastructure for laboratory monitoring and tele-monitoring, particularly in rural areas. Sufficient financial support and well-structured healthcare teams are essential to address these challenges and enhance the effectiveness of OPAT implementation [21].

### 3.7. China

The landscape of OPAT in China is uniquely shaped by strict regulatory interventions aimed at curbing the high rate of antimicrobial resistance. A defining policy is the “Outpatient intravenous (IV) Ban,” which prohibits the administration of intravenous antibiotics in the outpatient departments of secondary and tertiary hospitals across multiple provinces. Studies indicate that this ban has significantly reduced irrational antibiotic use but has also created a unique challenge: patients who genuinely require parenteral therapy are often diverted to the emergency department or inpatient ward [24]. To address this gap and meet the growing demand for aging-in-place, China has expanded home- and community-based care services nationwide. Among these, Home Infusion Therapy (HIT) is specifically designed for older adults and patients with complex chronic infections, with the goal of delivering value-based care while alleviating hospital overcrowding. However, despite initiatives such as hospital-at-home pilots, uptake remains limited due to insufficient infrastructure, variable service quality, and inadequate public awareness. Successful implementation of HIT will require a robust accreditation framework, standardized sterile compounding practices, integration of digital technologies, and comprehensive professional training to ensure patient safety and high-quality care [25]. China’s emerging home infusion therapy infrastructure may provide a potential platform for future OPAT development, although infection-specific OPAT pathways remain constrained by outpatient intravenous antibiotic restrictions and variable service capacity.

### 3.8. Hong Kong

Hong Kong’s OPAT model is structured. A major milestone in the region was the launch of the 6th Edition of the Interhospital Multi-disciplinary Programme on Antimicrobial ChemoTherapy (IMPACT) Guidelines in June 2025. This widely used antimicrobial prescribing guideline included a dedicated section on OPAT, providing clinicians with standardized protocols for patient selection and management [26]. The Hong Kong model emphasizes a multidisciplinary team (MDT) approach, where Infectious Disease (ID) specialists collaborate with ID nurses and pharmacists to mitigate risks such as adverse drug reactions (ADRs) and catheter-related complications [26].

### 3.9. Thailand

In Thailand, OPAT practice has been considered or implemented via the provision of continuous antimicrobial parenteral injections by hospitals admitting infectious patients [27], or at community hospitals as a community hospital-based parenteral anti-infective therapy (CohPAT) [28]. Its implementation improved the quality of antimicrobial use and patient outcomes. Appropriate antimicrobial dosing improved markedly, rising from 78% before implementation to 100% afterward (*p* < 0.001). In addition, the proportion of patients experiencing unfavorable outcomes, defined as treatment failure or in-hospital mortality, was significantly lower in the post-implementation group compared with the pre-implementation group (6% vs. 26%; *p* = 0.006). In light of the improved clinical outcomes and enhanced quality of care for septic patients following implementation, a pharmacist-led approach could be considered for adoption in OPAT settings [27].

### 3.10. Qatar

In Qatar, OPAT has evolved through both hospital-based and community-delivered models within the Hamad Medical Corporation (HMC) system. A major milestone was the launch of a dedicated OPAT service at the Communicable Disease Center in 2020, designed to allow patients requiring prolonged intravenous antimicrobial therapy to continue treatment in an outpatient setting rather than remaining hospitalized. The service operates seven days a week and is delivered by a multidisciplinary team comprising physicians, nurses, pharmacists, and patient educators, with active involvement of patients and families in decision-making. Common indications include pneumonia, skin and soft tissue infections, uncomplicated urinary tract infection with bacteremia, and meningitis [29]. In parallel, community-delivered OPAT has also been provided by paramedics and nurses from the national ambulance service with support from family physicians, reflecting a locally adapted model that extends parenteral antimicrobial care beyond hospital walls [30]. Recent data from a 320-bed general hospital in Qatar further support the feasibility of OPAT for selected patients with Gram-negative bacteremia. In a retrospective cohort of 125 adults treated between July 2021 and July 2022, patients completed therapy through inpatient intravenous treatment, a hospital-based OPAT IV room, home-based IV therapy, or early oral switch pathways. OPAT and oral step-down strategies were associated with shorter hospital stays, low readmission rates, and reduced drug and bed-day costs compared with continued inpatient care [31]. Nevertheless, the high prevalence of antimicrobial resistance, including ESBL-producing and multidrug-resistant organisms, remains an important challenge, underscoring the need for careful patient selection, microbiology-guided therapy, structured follow-up, and integration with antimicrobial stewardship programs [30].

### 3.11. Saudi Arabia

In Saudi Arabia, OPAT has gradually developed from institution-level initiatives into increasingly structured hospital- and home-based programs. One tertiary center established an OPAT program in November 2017 using both infusion clinic OPAT and home-infusion OPAT models, with the aims of standardizing outpatient parenteral antimicrobial administration, reducing hospitalization, improving bed turnover, and supporting safe antimicrobial delivery in the community. In a retrospective cohort of 90 patients treated between November 2017 and March 2020, all patients completed the intended antimicrobial course, with urinary tract infection, osteomyelitis, and bacteremia representing the most common indications. Ertapenem was the most frequently used agent, reflecting the high prevalence of ESBL-producing organisms, followed by vancomycin for Methicillin-resistant *Staphylococcus aureus* (MRSA)-related infections. The program was associated with low OPAT-related complication rates, avoidance of 1984 inpatient bed-days, and an estimated cost saving of 18 million Saudi riyals over the study period [32]. Another milestone was the implementation of the first OPAT program in Saudi Arabia and the Gulf region to utilize disposable elastomeric pumps. Initiated in May 2018, this multidisciplinary home-based model involved home medicine, pharmacy, nursing, and infectious diseases services. Between May 2018 and December 2019, 47 patients received 55 OPAT courses using 2869 elastomeric pumps, providing 927 days of home antimicrobial therapy, with no significant catheter-related complications or mortality reported [33]. More recently, a quality improvement project at one local hospital demonstrated the operational impact of OPAT clinics, reducing the proportion of prolonged-stay patients requiring intravenous antibiotics from approximately 23% to 12% during implementation and subsequently to 8%, while avoiding 673 hospital days and generating cost savings exceeding 2 million Saudi riyals [34]. Despite these favorable outcomes, OPAT implementation in Saudi Arabia remains largely institution-based, with variable service availability, limited national standardization, and antimicrobial stewardship concerns related to broad-spectrum agents. Future development should focus on national OPAT guidelines, standardized monitoring systems, and integration with antimicrobial stewardship programs.

### 3.12. Turkey

In Turkey, OPAT has been implemented mainly through hospital-based outpatient units, although its nationwide use remains limited and home-based or nursing center-based models are still underdeveloped. Early real-world experience from a 1000-bed teaching hospital demonstrated that OPAT could be both clinically effective and cost-saving within the Turkish healthcare system. In a retrospective cohort of 594 patients treated between October 2013 and December 2017, 98.5% achieved their end-of-treatment goals, while OPAT reduced costs to approximately 75% of equivalent inpatient parenteral antimicrobial therapy and saved 7078 inpatient bed-days, corresponding to 11.9 bed-days per patient [35]. Subsequent prospective data from Ankara between January 2019 and February 2021 further supported the safety and economic value of this model. Among 307 adults, urinary tract infection was the most common indication, followed by chronic osteomyelitis. Ertapenem was the predominant antimicrobial agent, reflecting the high burden of ESBL-producing Gram-negative infections, with ESBL positivity reported in 88.8% of Gram-negative isolates. The end-of-treatment success rate was 92.2%, drug-related adverse events occurred in 5.2% of patients, and 3040 bed-days were saved over 25 months. Cost analysis suggested that OPAT was approximately 50% less costly than inpatient treatment [36]. Pediatric OPAT experience in Turkey is more limited but emerging. A single-center pediatric study including 21 patients showed that pediatric OPAT (p-OPAT) shortened hospitalization by a median of 6 days, with no infusion-related adverse events; however, 14.3% required hospital readmission [37]. Taken together, these findings suggest that OPAT in Turkey is feasible, safe, and cost-effective for carefully selected adult and pediatric patients, but broader implementation will require standardized local or national guidelines, improved patient selection and follow-up criteria, and further development of home-based or community-supported OPAT models.

## 4. Discussion

Over the past decade, OPAT in Asia has evolved from predominantly ad hoc outpatient infusion practices toward more structured, multidisciplinary models. This transition has been most evident in settings with policy support, infectious disease specialist involvement, reimbursement mechanisms, and outcome-monitoring infrastructure [8]. The landmark multinational survey by Fisher et al. described OPAT in Asia as a “missed opportunity,” reflecting underdeveloped infrastructure and inconsistent delivery models [8]. However, recent developments indicate a clear paradigm shift, with several healthcare systems adopting structured OPAT systems supported by national policies, antimicrobial stewardship (AMS) frameworks, and healthcare system integration [16,26].

Reimbursement maturity tracks closely with programmatic structure: nationally funded schemes (Singapore’s MediShield Life; Taiwan’s 2025 NHI OPAT reimbursement) coincide with the most formalized governance and outcome registries, whereas settings without dedicated reimbursement (India, parts of South Korea) rely on ad hoc or institution-level financing and show weaker monitoring [19,21]. Governance models span infusion-center delivery (Singapore, Malaysia, South Korea), centrally coordinated multidisciplinary programs (Hong Kong, Taiwan), and home-based or hospital-at-home models supported by visiting nurses (Japan, Taiwan, China) [9,12,14,17,18,26]. Infectious diseases specialist oversight is a consistent feature of the more mature programs, while pharmacist-led models (Thailand’s CohPAT) demonstrate an effective alternative where physician capacity is limited [27,28]. Outcome-monitoring infrastructure is the most variable dimension, ranging from Singapore’s combined national registry to settings where follow-up and laboratory monitoring are incomplete in a substantial proportion of patients [8]. Finally, AMS integration is increasingly explicit in policy-driven programs but remains aspirational where broad-spectrum, once-daily agents are favored for administrative convenience. High rates of ESBL-producing and multidrug-resistant organisms (MDROs) warrant particular attention in several healthcare systems, including those in India, Qatar, Saudi Arabia, and Turkey (Table 2).

### 4.1. Clinical Outcome and Economic Benefits

Across the region, most reported readmission rates cluster around 5–10% where measured, comparable to Western benchmarks [39]. Broad-spectrum, once-daily agents, particularly ceftriaxone and ertapenem, predominate across multiple settings, reflecting a shared tension between administrative convenience and stewardship. Cost savings are consistently reported but expressed in heterogeneous units and timeframes, underscoring the need for standardized economic reporting (Table 3).

#### 4.1.1. Healthcare Resource Optimization

One of the most immediate and measurable advantages of OPAT is the reduction in inpatient bed utilization. In many Asian healthcare systems, hospital bed capacity remains constrained, particularly in tertiary referral centers. By enabling clinically stable patients to continue intravenous antimicrobial therapy outside the hospital setting, OPAT effectively alleviates bed shortages and improves patient flow. Studies from both Western and Asian contexts consistently demonstrate that OPAT can significantly reduce hospital length of stay without compromising clinical outcomes [13,15].

From an economic perspective, OPAT has been shown to be a cost-effective alternative to inpatient care. Cost analyses suggest that OPAT may reduce healthcare expenditures by 16–60% per treatment episode, with some Asian data indicating a cost reduction of over USD 1000 per patient compared with hospitalization [13,40,41]. These savings are largely driven by reduced inpatient costs, including accommodation, nursing care, and ancillary services. Importantly, cost-effectiveness extends beyond direct healthcare expenditure, as OPAT also facilitates earlier return to productivity for patients, which is particularly relevant in Asian societies where individuals often serve as primary income providers.

#### 4.1.2. Patient Outcomes and Safety

Beyond economic considerations, OPAT offers several clinical advantages. Home-based care reduces exposure to hospital-associated pathogens, thereby lowering the risk of nosocomial infections, including infections caused by MDROs [5]. This is particularly important in Asia, where antimicrobial resistance (AMR) rates are high [42]. Patient-centered outcomes also favor OPAT. Multiple studies have demonstrated improved quality of life and higher patient satisfaction associated with outpatient care models [4]. Patients benefit from increased mobility, psychological comfort, and the ability to maintain social and occupational roles. These factors contribute to improved adherence to therapy and overall treatment success.

### 4.2. Challenges in the Asian Context

Despite these benefits, several challenges remain that are unique to or particularly pronounced in the Asian context.

#### 4.2.1. Environmental and Drug Stability Considerations

One of the most under-recognized yet critical challenges in tropical and subtropical regions is antimicrobial stability. Many elastomeric infusion devices and antibiotic formulations have limited stability data at temperatures exceeding 25 °C. In regions where ambient temperatures frequently surpass this threshold, there is a risk of drug degradation, reduced antimicrobial efficacy, and potential treatment failure [43]. This issue underscores the urgent need for region-specific pharmacokinetic and stability studies. Without such data, clinicians may be forced to rely on less optimal antimicrobial regimens or restrict the use of continuous infusion strategies, thereby limiting the full potential of OPAT. Addressing this gap will require collaboration between academic institutions, pharmaceutical companies, and regulatory bodies.

#### 4.2.2. Financial and Regulatory Barriers

Financial and policy-related barriers remain significant obstacles to OPAT expansion in Asia. In some healthcare systems, reimbursement structures are still predominantly designed for inpatient care. As a result, transitioning patients to outpatient therapy may paradoxically reduce financial coverage, creating disincentives for both patients and providers. For example, insurance schemes in certain settings do not adequately cover outpatient intravenous therapy, while subsidies are often tied to hospitalization. This creates a structural misalignment between clinical best practices and financial incentives. Furthermore, the initial investment required to establish a comprehensive OPAT service, including trained personnel, infusion infrastructure, and monitoring systems, can be substantial, particularly in resource-limited settings [21].

Regulatory policies may also have unintended consequences. In China, restrictions on outpatient intravenous antibiotic use were implemented to curb inappropriate prescribing. While effective in reducing misuse, these policies have led to increased reliance on emergency departments or inpatient admissions for patients requiring parenteral therapy [24]. This highlights the need for balanced policies that promote appropriate use without restricting access to necessary care.

#### 4.2.3. Antimicrobial Stewardship Integration

Another critical challenge is the integration of OPAT into antimicrobial stewardship programs. Previous Good Practice Recommendations have highlighted the importance of antimicrobial stewardship in OPAT [39]. In practice, stewardship within structured OPAT programs is operationalized through several concrete mechanisms: mandatory infectious diseases review and formal sign-off before OPAT initiation; protocolized patient-selection and antimicrobial-choice criteria; scheduled review points for de-escalation and intravenous-to-oral switch; therapeutic drug monitoring for agents such as vancomycin and teicoplanin; defined treatment durations with pre-specified stop or review dates; regular multidisciplinary review of active cases; and routine capture of OPAT-specific stewardship metrics (e.g., proportion of broad-spectrum prescriptions, de-escalation rates, and days of therapy) [39]. Programs and policy frameworks in Singapore, Hong Kong, Taiwan, and Malaysia incorporate several of these elements through dedicated or multidisciplinary OPAT structures, while Thailand’s pharmacist-led model illustrates how protocolized pharmacist review can deliver measurable stewardship gains.

In unstructured OPAT settings, there is a tendency to prioritize convenience, adherence, and ease of home administration over optimal antimicrobial selection. As a result, once-daily, broad-spectrum agents such as ceftriaxone, ertapenem, or daptomycin may be favored, even when narrower-spectrum alternatives would be more appropriate. This concern is particularly relevant in several Asian settings, where ertapenem has been used preferentially for its convenient outpatient dosing, including for ESBL-related urinary tract infections in India and as a leading OPAT agent in cohorts from South Korea, Saudi Arabia, and Turkey. Such practice may increase selection pressure for carbapenem resistance. For example, one study reported that 46% of patients receiving self-administered OPAT were discharged on ertapenem and/or daptomycin specifically because of their once-daily dosing schedule [44], while another study suggested that 50% of ceftriaxone prescriptions in self-administered OPAT could have been rationalized, most commonly to penicillin [45]. Embedding OPAT within antimicrobial stewardship programs is therefore essential to ensure appropriate antimicrobial selection, promote carbapenem-sparing strategies, and preserve the practical advantages of outpatient delivery without unnecessarily increasing broad-spectrum antimicrobial exposure. Structured OPAT programs therefore provide an important opportunity to implement a “Start smart then focus” approach aligned with antimicrobial stewardship principles [46]. Initial empiric therapy can be rapidly reviewed and de-escalated based on microbiological results, clinical response, drug stability, patient preference, and service capacity. In addition, elastomeric pump–based continuous infusion may enable the use of time-dependent, narrower-spectrum antibiotics such as penicillin or cefazolin, thereby reducing unnecessary reliance on broad-spectrum once-daily agents [13]. Emerging OPAT strategies, including thrice-weekly teicoplanin, further illustrate how innovative dosing regimens may expand treatment feasibility while preserving stewardship goals [47]. Nevertheless, more outcome data are needed to determine whether manipulating dosing intervals or infusion strategies maintains clinical effectiveness and safety across different antimicrobial agents.

Oral step-down therapy is a complementary stewardship strategy that can substantially reduce intravenous exposure and line-related risk. Randomized evidence from the OVIVA trial in bone and joint infection and the POET trial in left-sided endocarditis demonstrated non-inferiority of appropriately selected oral regimens compared with prolonged intravenous therapy, supporting early, structured IV-to-oral switch where clinically appropriate as part of OPAT pathways [48,49]. Newer long-acting agents are also reshaping the OPAT landscape. The long-acting lipoglycopeptides dalbavancin and oritavancin permit single- or infrequent-dose treatment of Gram-positive infections, potentially obviating indwelling vascular access and daily infusions, while the once-weekly echinocandin rezafungin offers a comparable opportunity for selected invasive fungal infections [50,51,52]. A well-designed OPAT program should therefore balance feasibility, adherence, cost, and antimicrobial optimization, ultimately improving individual patient outcomes while contributing to broader efforts to combat antimicrobial resistance.

### 4.3. The Role of Innovation and “Local Champions”

The progress of OPAT in Asia over the past decade has been driven largely by “local champions”, clinicians and healthcare leaders who advocate for system change and develop context-specific solutions. These individuals play a pivotal role in establishing clinical workflows, standard operating procedures, and interdisciplinary collaboration. Concrete examples from the region illustrate this dynamic. In Singapore, sustained clinician-led advocacy and the development of a combined national outcomes registry underpinned the maturation of OPAT into a nationally integrated service [7,8]. In Hong Kong, the multidisciplinary IMPACT working group translated stewardship expertise into a dedicated, standardized OPAT section within a widely used prescribing guideline [26]. In Taiwan, infectious diseases specialists led the transition from emergency-department-initiated OPAT to a multidisciplinary, nationally reimbursed program and the integration of hospital-at-home services [15,16,17,18]. In Malaysia, the establishment of the first OPAT service at Hospital Sungai Buloh provided the local proof of concept that enabled subsequent post-pandemic national expansion [11], while in Thailand, a pharmacist-led model demonstrated how non-physician champions can drive measurable improvements in antimicrobial appropriateness and outcomes [27]. These examples underscore that local leadership, rather than any single delivery model, has been the common catalyst for OPAT development across diverse Asian settings.

#### 4.3.1. Strategic Partnerships

International collaboration is instrumental in accelerating OPAT development in Asia. Partnerships with established centers in Europe, North America, and Australia may facilitate knowledge transfer, training, and capacity building. This is particularly important in regions where infectious disease specialists are limited.

#### 4.3.2. Data Infrastructure and Outcome Monitoring

The transition from paper-based systems to electronic registries represents a major advancement in OPAT implementation. Robust data collection enables benchmarking, quality improvement, and demonstration of cost-effectiveness, critical factors for securing government and institutional support. Healthcare systems such as Singapore have demonstrated the value of centralized OPAT registries in driving policy development and resource allocation [7]. Digital health technologies, including telemedicine and remote monitoring systems, further enhance the safety and scalability of OPAT programs. These tools allow for real-time assessment of patient status, early detection of complications, and improved adherence to treatment protocols [53].

The expansion of OPAT registries also raises important governance considerations that must be addressed to ensure trust and sustainability. These include clear delineation of data ownership and stewardship responsibilities; robust protection of patient confidentiality in compliance with applicable data-protection legislation (such as Singapore’s and Taiwan’s Personal Data Protection Acts and China’s Personal Information Protection Law); appropriate regulatory and ethical oversight, including institutional data-governance committees and defined consent or waiver frameworks; and interoperability standards and data-sharing agreements that permit secure, de-identified benchmarking across institutions and, where feasible, across the whole healthcare systems. Establishing these governance structures at the outset, rather than retrofitting them, will be essential for any regional OPAT registry initiative.

### 4.4. Future Directions: Toward 2030

Several concrete, regionally specific priorities should guide OPAT development in Asia toward 2030. First, the establishment of a regional OPAT registry or network, potentially coordinated through existing societies, would enable benchmarking, multinational outcome comparison, and shared learning. Second, the development of Asia-specific OPAT consensus guidelines, adapted to regional pathogens, resource levels, and reimbursement structures rather than transplanted wholesale from Western recommendations, would support more consistent and locally appropriate practice. Third, adoption of standardized, core outcome reporting frameworks (e.g., hospitalization days avoided, readmission, adverse events, antimicrobial consumption, and patient-reported outcomes) would improve comparability across studies and jurisdictions. Fourth, dedicated stability and pharmacokinetic studies of antimicrobials and elastomeric devices under tropical and subtropical conditions are urgently needed, given that many existing stability data are generated at temperatures below those routinely encountered in much of Asia. Finally, robust economic evaluations, including cost-effectiveness and budget-impact analyses conducted specifically in low- and middle-income settings, are required to inform reimbursement decisions and ensure equitable access.

### 4.5. Limitations

Several limitations should be considered when interpreting this review. First, this is a narrative rather than a systematic review; although we applied a structured and transparent search strategy, study selection and synthesis remain subject to interpretive judgment. Second, much of the available evidence derives from single-center, observational, retrospective, or pilot studies, which limits internal validity and generalizability and precludes formal meta-analysis; outcome definitions (e.g., treatment success, readmission, and complications) and reporting periods were heterogeneous across studies, reducing comparability. Third, this review reflects OPAT practice in the twelve Asian healthcare systems for which published data could be identified and should not be interpreted as representing the full landscape of OPAT across Asia. Published implementation, outcome, and policy data were sparse or absent for many settings, including Central Asia, and several countries in East, South and Southeast Asia. The findings therefore characterize the better-documented programs rather than the totality of regional practice, and the absence of data from a given country should not be equated with the absence of OPAT activity. Fourth, there was limited and inconsistent reporting on the types and utilization of vascular access devices (e.g., peripheral cannulae, midline catheters, and peripherally inserted central catheters), which are central to OPAT safety and feasibility; this gap constrains conclusions regarding device-related complications and optimal access strategies. Finally, because OPAT policy and reimbursement in the region are evolving rapidly, some findings may be superseded by subsequent developments, and a degree of English-language and publication bias cannot be excluded.

## 5. Conclusions

OPAT in Asia is gradually moving beyond the “missed opportunity” described in 2017, although implementation remains uneven across healthcare systems. Over the past decade, the region has witnessed a transition from unstructured infusion practices to increasingly formalized, multidisciplinary care models that align with modern infectious disease management principles. The emergence of integrated OPAT systems, supported by antimicrobial stewardship and digital health innovations, represents a significant advancement in patient care. However, challenges remain, including drug stability in tropical climates, financial and regulatory barriers, and variability in implementation. Addressing these issues will require coordinated efforts across clinical, policy, and research domains. Continued investment in infrastructure, workforce development, and innovation will be essential to fully realize the potential of OPAT in improving clinical outcomes while optimizing healthcare resource utilization.

## Figures and Tables

**Table 1:** Comparison of OPAT models in twelve Asian healthcare systems.

	Primary Model	Key Features	Strengths	Challenges	Reference
Singapore	Infusion center and home-based care	National registry, strong policy support, MediShield coverage, elastomeric pump subsidy (2025)	Comprehensive OPAT bundle, robust data integration	High resource and cost requirements	[4,7,9,10]
Malaysia	Hospital-based infusion center	Established in 2016, expanded post-COVID-19, MDT approach	Standardized training, integration with AMS	Limited flexibility, predominantly hospital-centered	[11,12]
Japan	HaH and visiting nursing	Use of elastomeric pumps, preference for narrow-spectrum antibiotics	Supports antimicrobial stewardship, enables continuous infusion	Lack of unified national guidelines	[13,14]
Taiwan	ED-initiated OPAT and HaH (Expand to include discharged patients and outpatients)	National reimbursement including portable infusion devices (2025), MDT model, integration with HaH services	Cost-effective, high patient satisfaction	System still evolving	[15,16,17,18]
South Korea	Referral model and outpatient clinic	Widely used in tertiary centers	Reduces hospital length of stay	Inadequate monitoring and follow-up systems	[19,20]
India	Informal OPAT and pilot programs	High ESBL prevalence, frequent use of ertapenem	Significant cost-saving potential	Lack of standardization and infrastructure	[21,22,23]
China	HIT	“Outpatient IV antibiotic ban,” expansion of community/home care	Reduces inappropriate antibiotic use	Regulatory barriers limit OPAT expansion	[24,25]
Hong Kong	MDT-based OPAT	IMPACT guideline (2025), standardized protocols	High safety and standardization	Resource- intensive	[26]
Thailand	CohPAT	Pharmacist-led model	Improves antimicrobial use and outcomes	Limited scalability and standardization	[27,28]
Qatar	Hospital-based OPAT and community-delivered care	Dedicated Communicable Disease Center OPAT service (2020); 7-day MDT; ambulance-based community delivery	Multiple care pathways; shorter stay, low readmission, cost savings	High ESBL/MDR burden; needs structured follow-up and stewardship	[29,30,31]
Saudi Arabia	Infusion-clinic and home-infusion OPAT (elastomeric pumps)	Tertiary-center programs; first Gulf elastomeric-pump home OPAT	High completion rates; major bed-day and cost savings; low complications	Institution-based; limited national standardization; broad-spectrum AMS concerns	[32,33,34]
Turkey	Hospital-based outpatient OPAT unit (adult and pediatric)	Institution-based; emerging pediatric OPAT	High treatment success; substantial bed-day and cost savings	Limited national guidelines; broad-spectrum AMS concerns	[35,36,37]

Abbreviation: AMS, antimicrobial stewardship; ED, emergency department; MDT, multidisciplinary team; HaH, Hospital-at-home; HIT, Home Infusion Therapy; IMPACT, Interhospital Multi-disciplinary Programme on Antimicrobial ChemoTherapy; CohPAT, Community hospital-based parenteral anti-infective therapy; ESBL, extended-spectrum β-lactamase; MDR, multidrug-resistant.

**Table 2:** Thematic synthesis of OPAT programs in twelve Asian healthcare systems.

	Funding & Reimbursement	Governance & Coordination	ID Specialist Role & Delivery Model	Outcome Monitoring	AMS Integration
Singapore	Mixed public/private mandatory health savings account (MediShield Life; elastomeric pump subsidy, 2025)	Nationally integrated; strong policy support	ID-led MDT; infusion-center + home-based care	Combined national outcomes registry	Full OPAT bundle; stewardship-aligned
Malaysia	Public-hospital funded (MOH)	Hospital-based; standardized training	ID physician-led MDT (ID, pharmacist, nurse, AMO); infusion-center	Institution/hospital-level	Integrated with AMS
Japan	Home-care reimbursement	Not yet unified national protocol	Variable ID input; HaH + visiting-nurse, home-based	Institution/hospital-level	Narrow-spectrum via elastomeric pumps
Taiwan	National NHI OPAT reimbursement (2025)	MDT coordinated	ID specialist-led with dedicated case managers; ED-initiated + HaH (Expanded to include discharged patients and outpatients)	Evolving; HaH outcome data emerging	AMS via MDT and case managers
South Korea	Largely institution-based	Referral and outpatient-clinic models	Variable ID input; physician/clinic-based	Institution-level (≈25% follow-up missed; lab monitoring < 50%)	Advocated, not yet systematic
India	Largely institution-based	Informal and pilot programs	Limited ID capacity; mixed/informal	Limited (rural tele-monitoring gaps)	Limited; ertapenem predominant (high ESBL)
China	Constrained by outpatient IV ban; emerging HIT	Policy-restricted; community/home care	Variable; HIT, home/community-based	Limited, variable	Indirect (ban curbs misuse)
Hong Kong	Institutional/public	Highly structured MDT; IMPACT guideline (2025)	ID specialist-led with ID nurses, pharmacists; MDT	Standardized protocols	Embedded in IMPACT guideline
Thailand	Hospital/community-hospital based	CohPAT; pharmacist-led	Pharmacist-led with ID support; community-hospital model	Institution-level	Pharmacist-driven AMS (Increased dosing appropriateness)
Qatar	Public healthcare system (Hamad Medical Corporation)	Hospital-based (Communicable Disease Center) plus community/ambulance-service coordination	MDT (physicians, nurses, pharmacists, educators); hospital OPAT room + home/community delivery	Institution/hospital-level	Microbiology-guided therapy; AMS integration needed (high ESBL/MDR)
Saudi Arabia	Institution/hospital-based	Institution-based tertiary-center programs; limited national standardization	ID-inclusive MDT (home medicine, pharmacy, nursing, ID); infusion-clinic + home-infusion (elastomeric pumps)	Institution/hospital-level	Ertapenem-predominant; broad-spectrum/AMS concerns; integration needed
Turkey	Institution/hospital-based	Hospital-based outpatient units; limited nationwide coordination	ID clinic-based; hospital outpatient OPAT unit (adult + pediatric)	Institution/hospital-level	Ertapenem-predominant; broad-spectrum/AMS concerns; integration needed

Abbreviation: ID, infectious diseases; AMS, antimicrobial stewardship; MDT, multidisciplinary team; MOH, Ministry of Health; AMO, assistant medical officers; NHI, National Health Insurance; HaH, hospital-at-home; ED, emergency department; IV, intravenous; HIT, Home Infusion Therapy; CohPAT, Community hospital-based parenteral anti-infective therapy; ESBL, Extended Spectrum β-Lactamase.

**Table 3:** Comparative clinical and economic outcomes of OPAT in Asia (the content was compiled from multiple reported articles).

	Common Indications	Most-Used Antibiotics	Clinical Outcomes	Cost/Resource Outcome
Singapore	Orthopedic infections (40%)	Vancomycin (34%)	Readmission 8.9%; complications infrequent	Estimated cost savings SGD 207,200 across 51 patients in 2005
Malaysia	Bacteremia (53.5%); intra-abdominal abscess (30.2%)	Ceftriaxone (34.9%); ceftazidime (25.6%)	Readmission 9.3%	Limited published outcome data
Japan	Bacteremia (63.6%); Bone and joint infection (36.4%)	Cefazolin (39.4%); penicillin G (24.2%)	Readmission 4.5%	Reduction in medical expenses of USD 87,000 over 5.5 years for 66 patients
Taiwan	ED-initiated: UTI (30.3%); Musculoskeletal infection (29.5%) HaH: UTI (46.4%); Pneumonia (37.7%); Soft tissue infection (15.9%)	Per indication (MDT-guided)	Readmission 9.4–10.1%	8.9 hospital-days saved and NTD 34,367 cost reduction per patient
South Korea	UTI (27.3%); respiratory (20.8%); intra-abdominal (15.9%)	Ertapenem (26.0%); ceftriaxone (12.8%)	Readmission 12.2%	Limited published outcome data
India	UTI (30%); GI infections (20%)	Ceftriaxone (40%); Carbapenem (30%)	95% achieved afebrile status	Reduced hospital stay for 2 weeks
China	Chronic/complex infections (pilot HIT)	Restricted by outpatient IV ban	Limited published outcome data	Limited published outcome data
Hong Kong	Per IMPACT guideline	Protocolized (IMPACT)	Not specifically quantified	Not specifically quantified
Thailand	Lower respiratory tract (46%), urinary tract infection with bloodstream infection (12%)	Piperacillin/tazobactam (34.9%); meropenem (25.4%); vancomycin (11.1%)	Improved favorable outcomes (from 74% to 94%) after pharmacist-led implementation	Improved dosing appropriateness (from 78% to 100%) after pharmacist-led implementation
Qatar	UTI (36.7%)	Limited published antibiotics data, but high ESBL/MDRO prevalence	Readmission 7.6–30.7%	Reduction in medical expenses of 298 Qatari Riyals per patient
Saudi Arabia	UTI (26.9%); osteomyelitis (17.2%)	Ertapenem (43%); vancomycin (11.2%)	Read mission 11.2%	Reduction in medical expenses of SR 3,152,947 over 19 months for 47 patients
Turkey	UTI (48%); Bone and joint infection (16%)	Ertapenem (54%); Daptomycin (24%)	Readmission 7.2%	Reduction in medical expenses of 487 Turkish Liras per patient per day

Abbreviation: SGD, Singapore dollar; USD, United States dollar; ED, emergency department; UTI, urinary tract infection; MDT, multidisciplinary team; NTD, New Taiwan dollar; GI, Gastroenterological; HIT, Home Infusion Therapy; IV, intravenous; ESBL, Extended Spectrum β-Lactamase; MDRO, Multidrug-resistant organism.

## Data Availability

No new data were created or analyzed in this study. Data sharing is not applicable to this article.

## Supplementary Materials

The following supporting information can be downloaded at: https://www.mdpi.com/article/10.3390/antibiotics15070689/s1, Table S1. Characteristics of key Asia-specific studies and policy sources included in the review.

## Abbreviations

The following abbreviations are used in this manuscript:

ACAH	Acute Hospital Care at Home
ADRs	Adverse drug reactions
AMO	Assistant medical officers
AMR	Antimicrobial resistance
AMS	Antimicrobial stewardship
ASP	Antimicrobial Stewardship Programs
CDC	Centers for Disease Control and Prevention
CohPAT	Community hospital-based parenteral anti-infective therapy
COVID-19	Coronavirus disease 2019
ED	Emergency Department
ESBL	Extended-Spectrum Beta-Lactamase
GI	Gastroenterological
HaH	Hospital-at-home
HIT	Home Infusion Therapy
HITH	Hospital-in-the-Home
ID	Infectious Disease
MDR	Multidrug-resistant
MDRO	Multidrug-resistant organism
MDT	Multidisciplinary team
MOH	Ministry of Health
MRSA	Methicillin-resistant *Staphylococcus aureus*
NHI	National Health Insurance
NHIA	National Health Insurance Administration
NTD	New Taiwan dollar
OPAT	Outpatient Parenteral Antimicrobial Therapy
p-OPAT	Pediatric outpatient parenteral antimicrobial therapy
SGD	Singapore dollar
USD	United States dollar
WHO	World Health Organization
