# Supplementary material for: Outpatient Parenteral Antimicrobial Therapy in Asia: Evolution, Models, and Challenges over the Past Decade—A Narrative Review
Source: Antibiotics (Basel). 2026 Jul 15;15(7):689. doi: 10.3390/antibiotics15070689 (PMC13403625; doi:10.3390/antibiotics15070689)
Supplement: Supplementary file 1 [file antibiotics-15-00689-s001.zip › antibiotics-4397394-supplementary.pdf]

Supplementary Table S1. Characteristics of key Asia-specific studies and policy sources included in the review.

	Article (first author, year)	Design	Setting / population	Key findings relevant to OPAT	Evidence note
Cross-regional	Fisher et al., 2017	Multinational cross-sectional survey	171 facilities across 17 Asian countries	57% administered outpatient parenteral antibiotics; only 3% (excluding Singapore) had comprehensive specialist-led services; minimal outcome databases — basis for the "missed opportunity" framing.	Survey; broad coverage, self-reported
	Reidy et al., 2024	Comparative narrative review	Global OPAT	Highlights uneven global implementation and relative scarcity of published data outside early-adopter regions.	Narrative comparative review
Singapore	Fisher et al., 2006	Observational service evaluation	National OPAT service	Orthopedic infections 40%; vancomycin 34%; readmission 8.9%; estimated cost savings SGD 207,200 across 51 patients.	Single-system observational
	Wee et al., 2020	Observational (HRQoL)	Singapore OPAT patients	Improved health-related quality of life associated with OPAT outcomes.	Observational; patient-reported outcomes
	MOH elastomeric pump guidance, 2025	Policy / guideline document	Singapore	2025 recommendation to subsidise elastomeric infusion pumps for continuous outpatient/home infusion.	Authoritative policy
	MediShield Life coverage	Policy document	Singapore	OPAT services covered under MediShield Life; claim limit SGD 90/day.	Policy document
Malaysia	Afra Nahdia et al., 2018	Single-center observational	Hospital Sungai Buloh	Bacteremia 53.5%, intra-abdominal abscess 30.2%; ceftriaxone 34.9%, ceftazidime 25.6%; readmission 9.3%.	Single-center observational

	Lim et al., 2025	Mixed-methods evaluation	Malaysian public hospitals	Protocol to evaluate the national OPAT service; infusion-center MDT model with standardized training.	Study protocol
Japan	Hase et al., 2020	Single-center observational / descriptive	Tertiary care hospital	First comprehensive OPAT program; elastomeric pumps enabling narrow-spectrum agents (cefazolin, penicillin G); USD 87,000 cost reduction over 5.5 years for 66 patients.	Single-center observational
	Shibata et al., 2024	Interrupted time-series	Japan, home medical care	COVID-19 pandemic associated with increased home medical care utilization.	Population-level time-series
Taiwan	Chen et al., 2025	Retrospective cohort	ED-initiated OPAT	Saved 8.9 hospital days/patient; no increase in severe adverse events or 14-day readmission.	Single-center retrospective cohort
	NHIA Reimbursement, 2026	Policy document	Taiwan	National OPAT reimbursement program since August 2025; portable infusion devices included since November, 2025. Early Discharge Model under the Acute Hospital Care at Home Program since 2026.	Authoritative policy
	Tan et al., 2025	Observational	Hospital-at-home	Reduced hospital stay and medical expenditure with HaH/OPAT integration.	Observational
	Chou et al., 2025	Observational pilot cohort	HaH, nursing-home older adults	Early outcomes from the first HaH cohort; feasible with good satisfaction.	Pilot cohort
South Korea	Heo et al., 2023	Single-center retrospective observational	University-affiliated acute-care hospital	UTI 27.3%, respiratory 20.8%, intra-abdominal 15.9%; ertapenem 26.0%, ceftriaxone 12.8%; follow-up missed ≈25%; laboratory monitoring inadequate in >50%.	Single-center retrospective
	Kwon & Kim, 2024	Review / principles	Korea, antimicrobial stewardship	Advocates integrating OPAT into antimicrobial stewardship programs.	Narrative / review
India	Panda & Mathur, 2024	Narrative update	India	Structured OPAT remains rare; cultural and logistical barriers; need for standardized protocols and monitoring infrastructure.	Narrative review / update
	Kumar & Panda, 2025	Pilot longitudinal study	Resource-limited setting	UTI 30%, GI infections 20%; reduced hospital stay (≈2 weeks).	Pilot study

	Ramasubramanian et al., 2018	Observational comparative	ESBL acute pyelonephritis	Ertapenem OPAT effective and cost-saving for ESBL-producing Enterobacteriaceae pyelonephritis.	Observational comparative
China	Wang et al., 2020	Observational policy evaluation	Secondary/tertiary hospitals	Outpatient IV antibiotic ban reduced irrational use but diverted patients requiring parenteral therapy to ED/inpatient care.	Observational
	Zuo et al., 2026	Needs / demand assessment	China, home infusion therapy	Assesses need and demand for HIT in the aging population; identifies infrastructure gaps.	Needs-assessment / survey
Hong Kong	IMPACT 6th edition, 2025	Guideline document	Hong Kong	IMPACT 6th edition adds a dedicated OPAT section with standardized protocols; MDT (ID specialists, ID nurses, pharmacists).	Authoritative guideline
Thailand	Thomnoi et al., 2022	Pre–post implementation study	Community hospital (CohPAT)	Pharmacist-led CohPAT: appropriate dosing 78%→100% ($p<0.001$); unfavorable outcomes 6% vs 26% ($p=0.006$).	Single-center
	Thomnoi & Santimaleeworagun, 2021	Retrospective observational	Khlongluang Hospital	Describes the CohPAT model for continuous treatment of bacterial infections at a community hospital.	Observational
Qatar	HMC CDC OPAT service launch, 2020	Service announcement / institutional report	Qatar; Hamad Medical Corporation, Communicable Disease Center	Dedicated OPAT service launched (2020); seven-day MDT (physicians, nurses, pharmacists, patient educators); indications include pneumonia, skin and soft-tissue infection, UTI with bacteremia, and meningitis.	Institutional service announcement
	August et al., 2021	Retrospective cohort	Qatar; community-delivered OPAT via national	Paramedic- and nurse-delivered community OPAT with family-physician support; extends parenteral antimicrobial therapy beyond the hospital.	Retrospective cohort

			ambulance service		
	Chaponda et al., 2025	Retrospective cohort	320-bed general hospital; Gram-negative bacteremia	Compared inpatient IV, hospital OPAT IV room, home-based IV, and early oral switch; OPAT and oral step-down were associated with shorter stays, low readmission, and reduced drug and bed-day costs.	Single-center retrospective cohort
Saudi Arabia	Al Shareef et al., 2022	Retrospective cohort	Tertiary center; infusion-clinic and home-infusion OPAT	All patients completed therapy; UTI, osteomyelitis, and bacteremia most common; ertapenem most frequent (high ESBL), then vancomycin (MRSA); low complications; 1,984 bed-days avoided; ≈SAR 18 million saved.	Single-center retrospective cohort
	Zikri et al., 2021	Implementation / observational report	Tertiary teaching hospital; first Gulf-region elastomeric-pump home OPAT	2,869 elastomeric pumps providing 927 days of home antimicrobial therapy; MDT home-based model (home medicine, pharmacy, nursing, ID); no significant catheter-related complications or mortality.	Single-center implementation report
	De Asis et al., 2024	Retrospective quality-improvement project	Single hospital; prolonged-stay patients requiring IV antibiotics	OPAT clinic reduced prolonged-stay IV-antibiotic patients from 23% to 12% and then 8%; 673 hospital days avoided; >SAR 2 million saved.	Single-center retrospective investigation
Turkey	Bastug et al., 2021	Retrospective cohort	1000-bed teaching hospital	98.5% achieved end-of-treatment goals; OPAT cost ≈75% of inpatient parenteral therapy; 7,078 bed-days saved (11.9 per patient).	Single-center retrospective cohort
	Özbay et al., 2023	Prospective cohort	Single hospital in Ankara	UTI most common, then chronic osteomyelitis; ertapenem predominant (ESBL 88.8% of Gram-negative isolates); end-of-treatment success 92.2%; drug-related AE 5.2%; 3,040 bed-days saved; ≈50% less costly than inpatient.	Single-center prospective cohort
	Özbakır et al., 2026	Single-center pediatric observational	Pediatric OPAT (p-OPAT)	Shortened hospitalization by a median of 6 days; no infusion-related adverse events; 14.3% required readmission.	Single-center pediatric cohort

Abbreviations: AE, adverse event; CDC, Communicable Disease Center; CohPAT, community hospital-based parenteral anti-infective therapy; ED, emergency department; ESBL, extended-spectrum beta-lactamase; GI, gastrointestinal; HaH, hospital-at-home; HIT, home infusion therapy; HMC, Hamad Medical Corporation; HRQoL, health-related quality of life; ID, infectious diseases; IV, intravenous; MDT, multidisciplinary team; MOH, Ministry of Health; NHIA, National Health Insurance Administration; OPAT, outpatient parenteral antimicrobial therapy; SAR, Saudi riyal; UTI, urinary tract infection.
